# Hydrolysis and Methanogenesis in UASB-AnMBR Treating Municipal Wastewater Under Psychrophilic Conditions: Importance of Reactor Configuration and Inoculum

**DOI:** 10.3389/fbioe.2020.567695

**Published:** 2020-11-02

**Authors:** Judit Ribera-Pi, Antonio Campitelli, Marina Badia-Fabregat, Irene Jubany, Xavier Martínez-Lladó, Ewan McAdam, Bruce Jefferson, Ana Soares

**Affiliations:** ^1^Eurecat, Centre Tecnològic de Catalunya, Water, Air and Soil Unit, Manresa, Spain; ^2^Cranfield Water Science Institute, Vincent Building, Cranfield University, Cranfield, United Kingdom

**Keywords:** anaerobic membrane bioreactor, hydrolysis, municipal wastewater, psychrophilic temperature, upflow anaerobic sludge blanket, microbial community

## Abstract

Three upflow anaerobic sludge blanket (UASB) pilot scale reactors with different configurations and inocula: flocculent biomass (F-UASB), flocculent biomass and membrane solids separation (F-AnMBR) and granular biomass and membrane solids separation (G-AnMBR) were operated to compare start-up, solids hydrolysis and effluent quality. The parallel operation of UASBs with these different configurations at low temperatures (9.7 ± 2.4°C) and the low COD content (sCOD 54.1 ± 10.3 mg/L and pCOD 84.1 ± 48.5 mg/L), was novel and not previously reported. A quick start-up was observed for the three reactors and could be attributed to the previous acclimation of the seed sludge to the settled wastewater and to low temperatures. The results obtained for the first 45 days of operation showed that solids management was critical to reach a high effluent quality. Overall, the F-AnMBR showed higher rates of hydrolysis per solid removed (38%) among the three different UASB configurations tested. Flocculent biomass promoted slightly higher hydrolysis than granular biomass. The effluent quality obtained in the F-AnMBR was 38.0 ± 5.9 mg pCOD/L, 0.4 ± 0.9 mg sCOD/L, 9.9 ± 1.3 mg BOD_5_/L and <1 mg TSS/L. The microbial diversity of the biomass was also assessed. *Bacteroidales* and *Clostridiales* were the major bacterial fermenter orders detected and a relative high abundance of syntrophic bacteria was also detected. Additionally, an elevated abundance of sulfate reducing bacteria (SRB) was also identified and was attributed to the low COD/SO_4_^2–^ ratio of the wastewater (0.5). Also, the coexistence of acetoclastic and hydrogenotrophic methanogenesis was suggested. Overall this study demonstrates the suitability of UASB reactors coupled with membrane can achieve a high effluent quality when treating municipal wastewater under psychrophilic temperatures with F-AnMBR promoting slightly higher hydrolysis rates.

## Introduction

Low strength municipal wastewater is characterized by its low organic content (COD < 500 mg/L) and high solids content (TSS < 250 mg/L) (Henze et al., 2008). The most widely used technology for wastewater treatment is based on the activated sludge process, but this implies a high cost for aeration as well as the generation of high amounts of biomass (sludge) that needs to be further managed (Metcalf et al., 2003). In the recent years, anaerobic treatment of municipal wastewater has received much attention since it presents several advantages over aerobic processes. Anaerobic processes do not need forced aeration and the production of biogas makes this technology potentially self-sufficient in terms of energy. Moreover, anaerobic processes have a significant lower production of excess sludge. The upflow anaerobic sludge blanket (UASB) reactors, developed in the early 1970s by Lettinga et al. (1980) allowed the retention of high concentrated biomass thanks to the formation of a dense granular sludge. Since UASB reactors are fed in upflow mode they act as settling devices in which non-settable biomass is released and settable biomass is kept in the reactor. This characteristic allows the better exploitation of the reactor working volume (Metcalf et al., 2003). The use of UASB reactors for municipal wastewater treatment is common practice in tropical and semi tropical climates (Chong et al., 2012; Ozgun et al., 2013). However, the characteristics of municipal wastewater still constitute a challenge for anaerobic systems in temperate climates (Stazi and Tomei, 2018).

The key bottleneck of anaerobic processes under low temperatures (<20°C) is the reduction of the hydrolysis of particulate organic matter into soluble molecules, leading to an accumulation of suspended solids within the reactor, decreasing the efficiency of the overall process (Ozgun et al., 2013, 2015b; Petropoulos et al., 2017). Besides, it is difficult to achieve a low effluent chemical oxygen demand (COD) due to low substrate affinity of the anaerobic biomass (Ozgun et al., 2013). To overcome these limitations, anaerobic membrane bioreactor (AnMBR) technology has been investigated. The main success of AnMBRs for municipal wastewater treatment at low temperatures is the complete decoupling of hydraulic retention time (HRT) and sludge retention time (SRT). Hence, this configuration allows the complete retention of biomass inside the reactor and produces higher quality effluent in terms of COD, TSS, and pathogen counts (Liao et al., 2006). Furthermore, recent studies have shown how intermittent sparging can reduce the energy demand for controlling membrane fouling (Wang et al., 2018a). In the case of UASB configured AnMBR, the TSS in the membrane tank is lower than in CSTR configured AnMBR reactors, decreasing the fouling propensity of the membrane (Ozgun et al., 2015a).

Each stage of the anaerobic wastewater degradation process is executed by different microbial communities. The connections between microbial community structures and operational conditions have been studied (Ali Shah et al., 2014; Park et al., 2017; Svojitka et al., 2017; Zhu et al., 2017; Wang et al., 2018c). Microbial communities in anaerobic digesters have remained unknown for a long time (Morris et al., 2014). The recent application of molecular technologies, such as next-generation sequencing, has increased the knowledge and understanding of the complex microbial interactions in the anaerobic process (Fischer et al., 2016). While bacterial community structures and functions are known, with elevated functional redundancy despite variable taxonomic composition, numerous methanogen groups remain unidentified or poorly understood, and changes between digesters have not been examined in detail (Wilkins et al., 2015).

Anaerobic UASB reactors can use flocculent or granular biomass. From the superior settling capacity of granular sludge, it could be assumed that granular sludge could be advantageous for UASB based AnMBR. However, to date, few studies have compared granular and flocculent biomass with the purpose of evaluating the two inoculums in UASB configured AnMBR treating municipal wastewater. Martin Garcia et al. (2013) compared a granular UASB configured AnMBR with a flocculent CSTR configured AnMBR and confirmed the lower fouling propensity of the granular UASB while the biological performance was similar. Nevertheless, given the different reactor configuration applied, the impact of the flocculent or granular biomass in UASB configured AnMBR could not be directly inferred. On the other side Wang et al. (2019) compared granular and flocculent UASB configured AnMBRs concluding that flocculent biomass could be utilized as an alternative to granular biomass since similar permeability was obtained when sludge blanket was controlled. While Wang et al. (2019) focused their research in settleability of the particles exiting the sludge blanket, hydrolysis and microbial diversity still need to be investigated. Both the low temperature and the low COD content make the current work challenging and not previously reported. Thus, the aim of this work was to compare start-up, solids hydrolysis and effluent quality of three UASB configurations for municipal wastewater treatment under psychrophilic conditions (9.7 ± 2.4°C).

## Materials and Methods

### Experimental Set-Up

**FIGURE 1:**
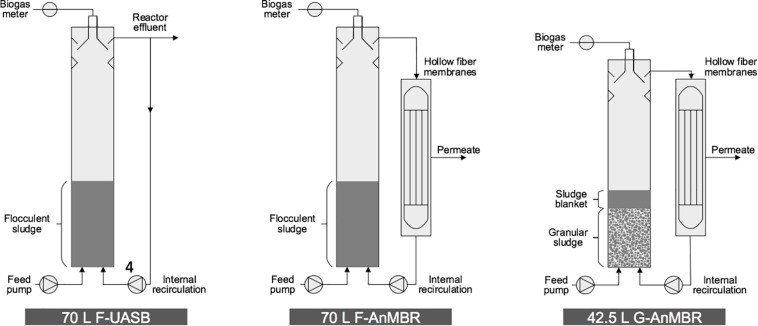
Schematic representation of pilot scale UASB with flocculent biomass (F-UASB), and 2 anaerobic membrane reactors with flocculent and granular biomass (F-AnMBR and G-AnMBR).

Three reactors were operated in parallel in this study; two 70 L cylindrical UASB (0.2 m diameter × 2.2 m height) and one 42.5 L UASB (0.19 m diameter × 1.5 m height) with lamella settlers for solid/liquid/gas separation at the top of the column (Figure 1). One of the 70 L reactor was operated as an UASB with flocculent biomass (F-UASB), while the other two reactors were operated as AnMBR but with flocculent and granular biomass (F-AnMBR and G-AnMBR) by coupling them to a submerged hollow fiber membrane. The flocculent 70 L reactors (F-AnMBR and F-UASB) were inoculated with 16 L of municipal digested sludge treating a mixture of primary and secondary sludges. The granular 42.5 L reactor (G-AnMBR) was inoculated with 16 L granular sludge from a mesophilic UASB used for pulp and paper industry. Both inoculums had a previous acclimation of 3 years treating the same wastewater and had been left without feeding for 5 months (Wang et al., 2018b). During the previous acclimation period, the effect on membrane permeability of peak flow was assessed.

Settled wastewater from Cranfield University wastewater treatment plant with a capacity of 2,840 population equivalent, was fed through the bottom of the three UASB reactors using peristaltic pumps (520U, Watson Marlow, Falmouth, United Kingdom). All three reactors were operated at an hydraulic retention time (HRT) of 8 h. Peristaltic pumps (620S, Watson 117 Marlow, Falmouth, United Kingdom) were used for the internal recirculation to keep the upflow velocity (Vup) at 0.4 m/h (Metcalf et al., 2014). In the three reactors, sludge expanded to about 30% of the column height. In the case of G-AnMBR, there was a sludge blanket layer above the granular sludge bed, which was composed of dispersed growth flocs from the influent, as previously described by Aiyuk et al. (2006) and Chong et al. (2012). Whilst for flocculent reactors there was no obvious differentiation between the sludge blanket and inoculum flocculent sludge bed. The sludge height in the UASB column was measured in a daily basis.

In the F-AnMBR and G-AnMBR configurations, the effluent was fed to 30 L membrane tanks and from there, recycled to the base of the reactor to maintain the upflow velocity. In F-AnMBR configuration, the hollow-fiber membrane module (ZW-10) (GE Water & Process Technologies, Oakville, Ontario, Canada) comprised four elements, each containing 76 polyvinylidene fluoride (PVDF) hollow fibers (0.52 m in length and 1.9 mm outer diameter), providing a total surface area of 0.93 m^2^. In G-AnMBR, the hollow- fiber membrane module (ZW-10) (GE Water & Process Technologies, Oakville, Ontario, Canada) comprised four elements, each containing 54 polyvinylidene fluoride (PVDF) hollow fibers (0.72 m in length and 1.9 mm outer diameter), providing a total surface area of 0.93 m^2^. The membranes had a nominal pore size of 0.04 μm.

Permeate was driven using peristaltic pumps (520U, Watson Marlow, Falmouth, United Kingdom). In F-AnMBR, transmembrane pressure was monitored by a pressure transducer (−1 to 1 bar, Gems sensor, Basingstoke, United Kingdom) in the permeate line and recorded by a data logger (ADC-2006, Pico Technology, St Neots, United Kingdom). In the G-AnMBR, pressure transducers on the permeate line (−1 to 1 bar, PMC 131, Endress + Hauser, Manchester, United Kingdom) and at the bottom of the membrane tank (0–2.5 bar, 060G2418, Danfoss, Nordborg, Denmark) were used to monitor TMP and liquid level height, respectively.

Nitrogen-enriched air, produced by a nitrogen generator (NG6, Noblegen gas generator, Gateshead, United Kingdom), was used for continuous gas sparging. Specific gas demand per surface area (SGDm) of 2.0 m^3^/(m^2^ h) was kept along operation. Since the HRT was fixed to 8 h, it resulted in an initial normalized permeate flux of 13.2 LMH for F-AnMBR and 8.3 for G-AnMBR, normalized to 20°C according to Judd (2011):

$$J_{T}=J_{20}\cdot 1.025^{\left(T-20\right)}$$

### Analytical Methods

Alkalinity, pH, total suspended solids (TSS) and biological oxygen demand (BOD_5_) were measured according to standard methods (APHA et al., 2012). Sulfate concentration, total and soluble chemical oxygen demand (COD) were analyzed with Merck test kits (Merck KGaA, Darmstadt, Germany). Soluble COD was measured after filtering with 0.45 μm retention membrane filters (47 mm Cellulose Nitrate Membranes, Whatman, GE Healthcare Life Sciences, Little Chalfont, United Kingdom). Particle size distribution (PSD) was measured using Mastersizer 3000 laser diffraction particle size analyzer (Malvern Instruments Ltd., Malvern, United Kingdom). Samples for volatile fatty acids (VFAs) analysis were filtered (0.45 μm), acidified (H_2_SO_4_) and kept frozen at −20°C prior to its analysis. VFAs were quantified using high performance liquid chromatography (HPLC) by means of a Shimadzu HPLC system (Kyoto, Japan) with a Phenomenex Rezex ROA/Organic Acid 7.80 mm × 300 mm column (Phenomenex, Macclesfield, United Kingdom) according to Parawira et al. (2004). Biogas flow rate was measured by means of three gas meters (TG0.5, Ritter, Bochum, Germany). Biogas methane (CH_4_) composition was analyzed by a gas analyzer (Servomex 1440, Crowborough, United Kingdom). Dissolved methane was calculated using unitless form of Henry’s law for dissolved gases, which, in the reactor headspace will depend on temperature, partial pressure and solubility (Crone et al., 2016). The unitless form of Henry’s law is described in Eq. 1.

(1) $$\frac{C_{g}}{C_{s}}=H_{u}$$

Where Cg is the concentration of constituent in gas phase (mg/L), Cs is the saturation concentration of constituent in liquid (mg/L) and Hu is unitless Henry’s law constant, which will vary with temperature (Metcalf et al., 2014). Methane yield was calculated accounting for COD used for methanogenesis, i.e., discounting the COD required to reduce 100% of the sulfate available for the sulfate reducing bacteria (Lens et al., 1998), and considering the total methane produced (gaseous and dissolved methane contents). The percentages of hydrolysis and methanogenesis were calculated according to the Eqs. 2 and 3, presented by Elmitwalli et al. (2002).

(2) $$\mathrm{Hydrolysis}\left(\%\right)=100\times \frac{{\mathrm{CH}}_{4}\,\mathrm{as}\,\mathrm{COD}+{\mathrm{sCOD}}_{\mathrm{eff}}-{\mathrm{sCOD}}_{\inf}}{{\mathrm{tCOD}}_{\inf}-{\mathrm{sCOD}}_{\inf}}$$

(3) $$\mathrm{Methanogenesis}\left(\%\right)=100\times \frac{{\mathrm{CH}}_{4}\,\mathrm{as}\,\mathrm{COD}}{{\mathrm{tCOD}}_{\inf}}$$

In order to evaluate the differences in the measured parameters Tukey HSD tests for multiple comparison of means were performed (*p* < 0.05), whereby different subscript letters indicate statistically significant differences.

### Microbial Community Analysis

#### Sludge Sampling, DNA Extraction, and Library Preparation

Biomass samples from the three reactors were taken after 45 days of operation. Samples were frozen at −80°C for further analysis. For DNA extraction, samples were centrifuged at 5,000 × g for 10 min. DNA extraction from the obtained pellet was performed using PowerSoil^®^ DNA Isolation Kit (Mo Bio Laboratory Inc., United States). Library preparation was performed at the Centre for Omic Sciences, COS (Reus, Spain). Partial bacterial 16S rRNA gene sequences were amplified from extracted DNA using the primer pair 341F-532R (5′-CCTACGGGRSGCAGCAG-3′; 5′-ATTACCGCGGCTGCT-3′), which targets the V3 region of the 16S rRNA gene sequence and primer pair 515F-806R (5′-GTGCCAGCMGCCGCGGTAA-3′; 5′-GGACTACHVGGGTWTCTAAT-3′) which targets the V4 region. Partial archaeal 16s rRNA gene sequence was amplified using the primer pair S-D-Arch-0787-a-S-20 and S-D-Arch-1043-a-A-16 (5′-ATTAGATACCCSBGTAGTCC-3′; 5′-GCCATGCACCWCCTCT-3′) (Fischer et al., 2016). All these primers were designed to include at their 5′ end one of the two adaptor sequences used in the Ion Torrent sequencing library preparation protocol linking a unique Tag barcode of 10 bases to identify different samples. PCR cycle parameters are described elsewhere (Ellis et al., 2012; Milani et al., 2013; Tridico et al., 2014; Fischer et al., 2016). In short, PCR products were confirmed by a 2% agarose gel and specific bands were excised and then purified using Nucleospin Gel (Macherey-Nagel, Germany). The concentration of the PCR amplicons was analyzed by electrophoresis on an Agilent 2100 Bioanalyzer (Agilent Technologies, United States) and the kit Agilent High Sensitivity DNA (Agilent Technologies, United States). Equimolar pools (60 pM) of each fragment and sample were combined.

#### Ion Torrent PGM Sequencing and Sequenced-Based Microbiome Analysis

Multiplexed samples were prepared for sequencing employing the Ion 520 and Ion 530 Kit-Chef (Life Technologies, United States) according to the manufacturer’s instructions. Prepared samples were loaded on an Ion 530 Chip and then sequenced using an Ion GeneStudio S5 (Life Technologies, United States) at 850 reads per run. After sequencing, individual sequence reads were filtered by the PGM software to remove low quality and polyclonal sequences. Those reads were analyzed using QIIME (v1.9.1) (Caporaso et al., 2011), the analysis included OTU clustering, Alpha-diversity analysis, OTU analysis and species annotation. The OTU assigning method was UCLUST and the taxonomy assigning method was BLAST. The sequence similarity threshold for both OTU and taxonomy assignments was 97%. The taxonomy database employed was *GreenGenes* for 16s rRNA gene sequences. Principal component analysis (PCA) was used to compare reactors microbial communities. PCA was performed using Matlab.

## Results and Discussion

### Performance of UASB and UASB-AnMBR Systems

The influent, effluent and membrane permeates characteristics are presented in Table 1. In this study an initial acclimation period took place from day 0–16 at a temperature of 7.1 ± 1.9°C, followed by a steady state period (days 17–45) which was conducted at 10.3 ± 2.1°C which represented a significantly higher temperature (Table 1). The average temperature of this study was lower than temperatures of previous AnMBR studies for the treatment of municipal wastewater, Gouveia et al. (2015) operated at 18 ± 2°C, Wang et al. (2018b) at 16.3 ± 3.7°C, Martin Garcia et al. (2013) worked in a range of 10–20°C and Fawehinmi et al. (2007) at 12 ± 0.5°C. The settled municipal wastewater presented an average COD content of 153 ± 75.1 mg/L, representing a low strength wastewater for anaerobic processes (Stazi and Tomei, 2018). Previous studies with settled municipal wastewater presented equal to higher COD contents from 221 to 976 mg/L showing it is possible to use AnMBR technology for the treatment of this low strength wastewater (Martin Garcia et al., 2013; Shin et al., 2014; Gouveia et al., 2015; Wang et al., 2018a,b). Thus, both the low temperature and the low COD content, made the current work challenging and not previously reported.

**TABLE 1:** Inlet wastewater and effluent characteristics (average ± standard deviation).

Parameter	Inlet	F-UASB			F-AnMBR					G-AnMBR				
				
		Reactor effluent			Reactor effluent		Permeate			Reactor effluent		Permeate		
						
		Day 0–16	Day 17–45	Day 0–45	Day 0–16	Day 17–45	Day 0–16	Day 17–45	Day 0–45	Day 0–16	Day 17–45	Day 0–16	Day 17–45	Day 0–45
Temp. (°C)	9.7 ± 2.4	7.1 ± 1.9	10.3 ± 2.1	9.3 ± 2.4	7.1 ± 1.9	10.3 ± 1.9	n.a.	n.a.	n.a.	7.1 ± 1.9	10.3 ± 2.0	n.a.	n.a.	n.a.
pH	7.8 ± 0.2	8.1 ± 0.2	8.0 ± 0.1	8.0 ± 0.1	8.0 ± 0.2	7.9 ± 0.1	8.6 ± 0.2	8.7 ± 0.1	8.7 ± 0.1	8.1 ± 0.2	8.3 ± 0.1	8.6 ± 0.1	8.7 ± 0.1	8.7 ± 0.1
sCOD (mg/L)	54.1 ± 10.3	42.6 ± 19.3	53.4 ± 6.3	49.5 ± 13.3	58.7 ± 22.3	56.7 ± 8.3	39.9 ± 7.0	38.0 ± 5.9	38.1 ± 6.0	68.0 ± 23.1	59.5 ± 9.0	39.1 ± 6.7	28.4 ± 4.2	31.8 ± 6.7
pCOD (mg/L)	84.1 ± 48.5	49.8 ± 16.8	36.0 ± 16.7	39.4 ± 19.4	86.8 ± 62.6	182 ± 93.8	1.3 ± 1.3	0.4 ± 0.9	0.7 ± 1.1	443 ± 152	266 ± 106	1.5 ± 0.8	0.7 ± 1.3	0.8 ± 1.4
BOD*5* (mg/L)	67.8 ± 25.7	71.6 ± 27.9	68.2 ± 11.4	69.9 ± 17.5	107	103 ± 12.4	10.9	9.9 ± 1.3	11.1 ± 1.9	143 ± 73.5	131 ± 32.8	13.2 ± 6.9	5.9 ± 0.2	6.5 ± 1.2
TSS (mg/L)	47.7 ± 30.1	13.1 ± 14.0	27.2 ± 14.8	22.0 ± 15.7	29.3 ± 18.9	121 ± 58.4	<1	<1	<1	238 ± 109	173 ± 60.6	<1	<1	<1
Alkalinity (mg CaCO*3*/L)	226 ± 20.7	310 ± 7.1	291 ± 18.1	299 ± 16.9	328 ± 17.7	258 ± 31.9	335 ± 7.1	264 ± 9.5	292 ± 38.1	348 ± 10.6	316 ± 29.6	345 ± 0.0	304 ± 26.7	321 ± 29.3
VFA (mg/L)	8.1 ± 7.9	n.a.	0.4 ± 0.7	n.a.	n.a.	n.a.	n.a.	3.6 ± 4.4	2.6 ± 4.4	n.a.	n.a.	n.a.	2.0 ± 2.4	2.0 ± 2.4
SO_4_^2–^ (mg/L)	70.0 ± 1.5	n.a.	n.a.	n.a.	n.a.	n.a.	n.a.	n.a.	n.a.	n.a.	n.a.	n.a.	n.a.	n.a.

*n.a., not analyzed.*

Inlet pH was slightly alkaline 7.8 ± 0.2 and it did not vary significantly after treatment (Table 1). However, a pH increase was observed in the permeate after membrane filtration similarly to observations of Wang et al. (2018a). This increase is congruent with to CO_2_ stripping due to the continuous nitrogen gas sparging which adjusted the carbonate-bicarbonate buffer equilibrium toward higher pH values. The average BOD_5_ content in the feed wastewater was 67.8 ± 25.7 mg/L. F-UASB treatment showed a low BOD_5_ removal (8.6 ± 7.7%), while for F-AnMBR and G-AnMBR sensibly higher removal percentages were observed, specifically 80 ± 5.9% and 89 ± 4.3% respectively. For AnMBR configurations, permeate COD and BOD_5_ obtained are comparable to previous studies of AnMBR operated on the same sewage (Martin Garcia et al., 2013; Wang et al., 2018a).

**FIGURE 2:**
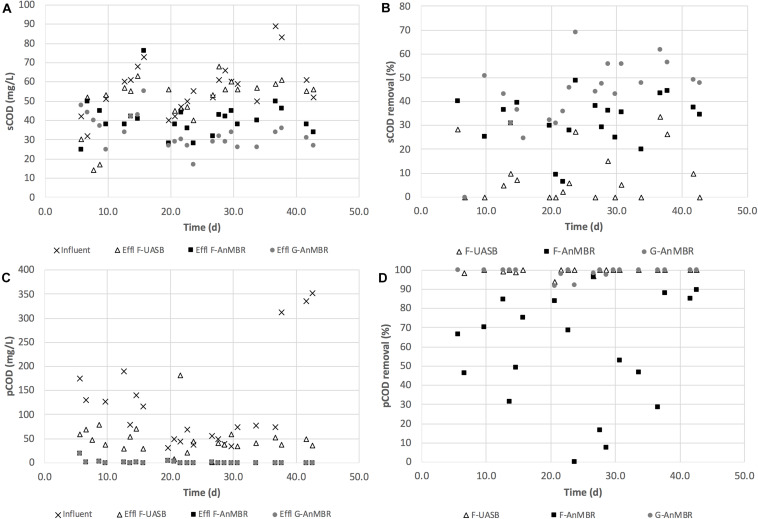
**(A)** sCOD (mg/L); **(B)** sCOD removal (%); **(C)** pCOD (mg/L); and **(D)** pCOD removal (%) for F-UASB, F-AnMBR and G-AnMBR.

COD removal efficiency remained stable from the beginning of the operation as can be observed from Figure 2, achieving, during the acclimation period (days 0–16), sCOD (F-UASB—13 ± 11%, F-AnMBR—35 ± 6%, G-AnMBR—31 ± 18%) and pCOD (F-UASB—61 ± 19%, F-AnMBR—99 ± 1%, G-AnMBR—100 ± 0%) removals similar to those obtained for the rest of the period studied (days 17–45). sCOD removal from day 17–45 was (F-UASB—11 ± 12%, F-AnMBR—31 ± 12%, G-AnMBR—48 ± 11%) while for pCOD it was (F-UASB—60 ± 32%, F-AnMBR—99 ± 2%, G-AnMBR—98 ± 2%). The quick start-up, according to COD removal efficiencies during acclimation period (days 0–16) is attributed to the previous acclimation of the biomass to the temperate treatment conditions, even with the previous 5-month period of storage without feeding. For the whole operation period, average sCOD removal in the F-UASB without the membrane was 11.0%, varying from 1 to 28%, while for the AnMBR configurations sCOD removal was around 32 ± 11% for the flocculent sludge and 43 ± 15% for the granular sludge reactor (Figure 2). Higher sCOD removals were obtained when using membrane configurations. The pore size of the filter used for sCOD determination was 0.45 μm while the average pore size of the membrane was 0.04 μm. This indicated that an important fraction of soluble COD would be retained by the ultrafiltration membrane (Gouveia et al., 2015). The same was observed previously by Ozgun et al. (2015a) when comparing UASB and AnMBR performances. Similar sCOD removal efficiencies were observed for the AnMBRs, although a slightly better removal efficiency was observed for G-AnMBR. During the whole period, AnMBR configurations, as expected, achieved high pCOD removals for both sludge types, accounting for 99 ± 1% in both cases (Figure 2). In comparison, although the F-UASB was capable of partially removing pCOD (57 ± 30%), its efficiency was lower than the AnMBRs due to the solid retention capacity of the membranes. Similarly, Hejnic et al. (2016) reported an increase from 64 to 85% in the total COD removal after adding a membrane to a UASB system. Also, Peña et al. (2015) demonstrated that membrane effect increased 45% total COD removal efficiency.

**FIGURE 3:**
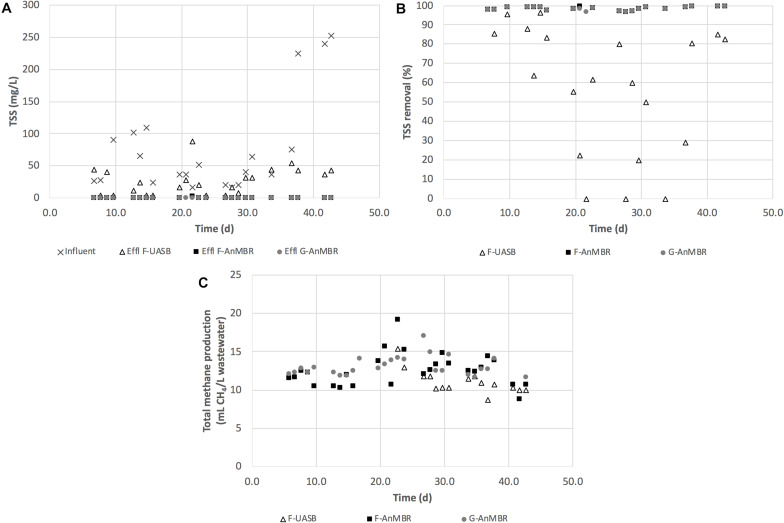
**(A)** TSS (mg/L); **(B)** TSS removal (%); and **(C)** total methane production (mL CH_4_/L wastewater) for F-UASB, F-AnMBR, and G-AnMBR.

After the acclimation period, the methane yields of the three configurations were compared, including both gas and dissolved methane contents (Figure 3). The contribution of the dissolved methane, in relation to the total methane production, was very significant in all configurations: 89 ± 9.4% in the F-UASB, 85 ± 12% in the F-AnMBR and 80 ± 21% in the G-AnMBR. Clearly demonstrating the need to recover the dissolved fraction in systems operated at low temperatures. The F-UASB methane yield (0.13 ± 0.12 m^3^ CH_4_/kg tCOD removed) was lower than that for the F-AnMBR (0.20 ± 0.14 m^3^ CH_4_/kg tCOD removed) and G-AnMBR (0.18 ± 0.09 m^3^ CH_4_/kg tCOD removed) although high standard deviations were obtained for this parameter. The difference was attributed to the rejection properties of the membrane that retains all particles, colloids and macromolecules which could then be utilized for methane production. Although the methane yield was higher in the anaerobic systems with membrane filtration, the potential energy production in the F-UASB reactor was also significant, but in this case with theoretically less capital (e.g., no membrane filtration tank) and operational costs (e.g., no need for membrane sparging). Nevertheless, the anaerobic configurations with membrane produced a higher effluent quality that can be discharged for fertigation uses, for example, without the need for further treatment. Hence, the economic and environmental sustainability of these systems needs to be further investigated to draw conclusions on their overall suitability for application in WWTPs from a holistic point of view.

Membrane bioreactors are widely known for their efficient retention of particulate matter (Chang, 2014). As expected, TSS removal in the configurations including a membrane were higher than for the F-UASB not coupled to a membrane, with observed removal levels of 99 ± 1% in F-AnMBR and G-AnMBR, and 57 ± 34% in F-UASB (Figure 3). Inclusion of the membrane resulted in an accumulation of solids within the membrane tank that exceeded the levels observed in the F-UASB effluent (Table 1). This could be explained by the build-up of sludge and suspended particles into the AnMBR systems thanks to the complete retention of particles by membranes that, in the case of F-UASB were cleared from the system.

Turbulence created by the gas sparging in the membrane tank could also lead to particle break-up and disintegration that would also be retained in the system. This effect was previously described at lab scale by Ozgun et al. (2015b) when comparing UASB and AnMBR performances. In spite of the increase of effluent solids, the UASB reactor still acts as a proper biofilter prior to membrane treatment, which prevents the membrane from being exposed to elevated solids concentrations. Although TSS were higher for AnMBR than for UASB, its concentration was still kept at <500 mg/L, which is lower than the concentrations faced by membranes in CSTR-based AnMBRs (Martin Garcia et al., 2013).

**FIGURE 4:**
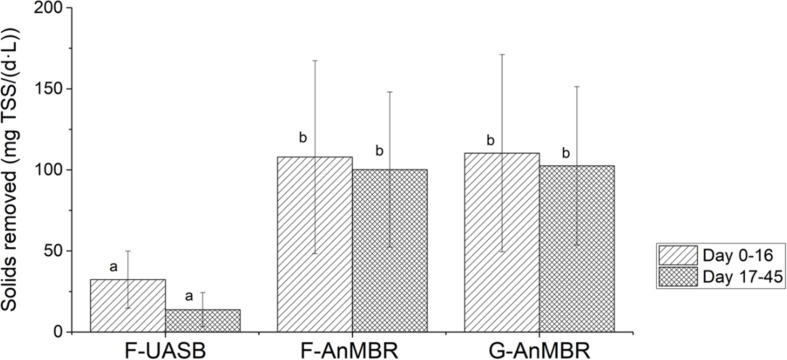
Total suspended solids mass balance. Error bars represent standard deviation. Different subscripts represent the statistically differences using Tukey HSD comparisons (*p* < 0.05).

Figure 4 shows the total suspended solids mass balance for the three studied configurations. As the membrane retains all solids, the solids removal rate is defined by the influent solids rate whereas the UASB removal rate is impacted by the influent loading rate. The mass balance for F-AnMBR and G-AnMBR was statistically similar, meaning biomass inoculum did not affect the TSS removal efficiency of the system. From these results it can be stated that the membrane becomes essential when it comes to TSS removal, since F-UASB TSS removed per day and per volume of reactor (27 ± 20 mg TSS/d.L) were significantly lower than the AnMBR ones (111 ± 57 mg TSS/d.L) for F-AnMBR and 113 ± 59 mg TSS/d.L for G-AnMBR).

Particle size analysis were performed on a daily basis and they showed no big differences between flocculent reactors in which median particle size, D50, was 66.8 ± 61.1 μm and 78.5 ± 75.6 μm for F-AnMBR and F-UASB respectively while D90 was 280 ± 106 μm for F-AnMBR and 302 ± 107 for F-UASB. The particle size distribution observed from the granular AnMBR was shifted toward smaller particle sizes compared to those observed from the flocculent reactors, with a D50 of 21.1 ± 11.1 μm and a D90 of 139 ± 78.8 μm. As previously observed for the TSS values, the operation of the AnMBR did not allow the wash out of finer particles and this was reflected in the PSD. The most important observed differences in the PSDs were related to the reactor biomass. Ozgun et al. (2015a) compared a flocculent UASB reactor before and after membrane addition, concluding that the membrane incorporation induced a decrease in particle size distribution (PSD) and a drop-in sludge settleability while no decrease in permeate quality was observed, which is in agreement with the results obtained.

**FIGURE 5:**
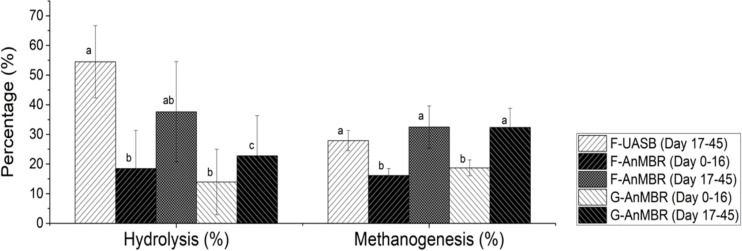
Percentage of hydrolysis and methanogenesis. Error bars represent standard deviation. Different subscripts represent the statistical differences using Tukey HSD comparisons (*p* < 0.05).

Hydrolysis has been demonstrated to be the limiting step in anaerobic processes at low temperatures rather than methanogenesis, since methanogenesis is less temperature sensitive than hydrolysis (Lester et al., 2009; Petropoulos et al., 2017). From results shown in Figure 5, there was no big differences between hydrolysis and methanogenesis under the working conditions tested. After the acclimation period, hydrolysis was statistically similar for F-UASB (54 ± 12%) and F-AnMBR (38 ± 17%) while it was lower for G-AnMBR (23 ± 14%) (Figure 5). It can be stated that flocculent sludge seemed to perform better for hydrolysis step than the granular sludge. However, no differences were observed in methanogenesis after the acclimation period since it was statistically similar for F-UASB (28 ± 3%), F-AnMBR (33 ± 7%) and G-AnMBR (32 ± 6%).

**FIGURE 6:**
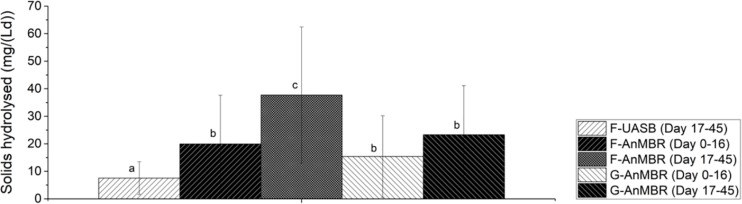
Solids hydrolyzed in the different reactors over specific time intervals. Error bars represent standard deviation. Different subscripts represent the statistically differences using Tukey HSD comparisons (*p* < 0.05).

To further evaluate differences between the three configurations tested, the efficiency of solids hydrolysis in terms of the mass of solids hydrolyzed per volume and time was calculated and it is shown in Figure 6. From this, it can be stated that, after the acclimation period, the higher solid hydrolysis was observed in the F-AnMBR configuration (38 ± 25%), compared to the F-UASB (8 ± 6%) and G-AnMBR (23 ± 18%). Given that in UASB reactors settled biomass acts as a filter, it was hypothesized that granular sludge could act as a coarse filter while flocculent sludge as a fine filter. This differences in particle size in the biomass and thus its filtration performance, could explain the differences in the efficiency of solids hydrolysis in terms of mass solids hydrolyzed per volume and time (Wang et al., 2019). As can be shown in Table 1, TSS in the granular reactor effluent (173 ± 60.6 mg/L) were higher than for the flocculent one (121 ± 58.4 mg/L).

### Microbial Community Structure

Microbial community analyses were performed. All the rarefaction curves showed gentle slopes under current sequencing depth indicating that the sequencing libraries could properly reflect the microbial communities. Alpha-diversity analysis revealed greater richness and diversity values in the bacterial community compared to the archaeal community, which is consistent with previous studies of microbial communities in AnMBR (Seib et al., 2016; Table 2). Chao 1 and Shannon Indexes indicated that bacterial diversity was higher in F-AnMBR, while archaeal diversity was higher in F-AnMBR and G-AnMBR than in F-UASB when analysing 16S rRNA.

**TABLE 2:** Characteristics of sequencing libraries.

Target gene	Sample	Number of sequences	Number of OTUs	Chao 1 value	Shannon index
Bacterial 16S rRNA (V3 + V4)	F-UASB	239,419	4,187	4303	9.41
	F-AnMBR	294,431	4,181	4290	9.50
	G-AnMBR	153,641	3,345	3828	9.08
Archaeal 16S rRNA	F-UASB	161,342	419	430	3.31
	F-AnMBR	151,423	438	455	3.50
	G-AnMBR	209,826	440	457	3.54

**FIGURE 7:**
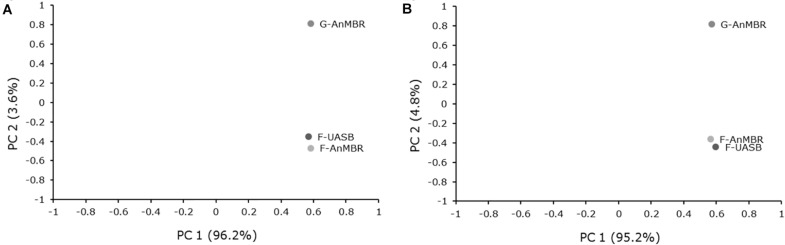
Principal component analysis (PCA) of **(A)** Bacteria and **(B)** Archaea 16S rRNA sequencing profiles for each reactor.

PCA analysis demonstrated that the three samples analyzed were highly similar (Figure 7). The three samples were clustered near the same value for first principal component (PC 1), which explained >95% of the variance for both analyses (bacteria and archaea). Main differences between reactors were due to second principal component (PC 2) which explained less than 5% of the variance. As can be inferred from PCA results, higher similarities were found in flocculent reactors, regardless of the membrane presence in the system. Thus, reactor inoculum had a higher influence in microbial community than reactor configuration. As the same wastewater was fed in the three reactors and the working temperature was the same in all cases, it could explain high similarity between samples. As commented before, this start-up was performed with a seed sludge which had been previously acclimated for 3 years treating the same wastewater although it had been left without feeding for 5 months before the operation commenced. This fact is consistent with the low variability of microbial communities in the three reactors.

**FIGURE 8:**
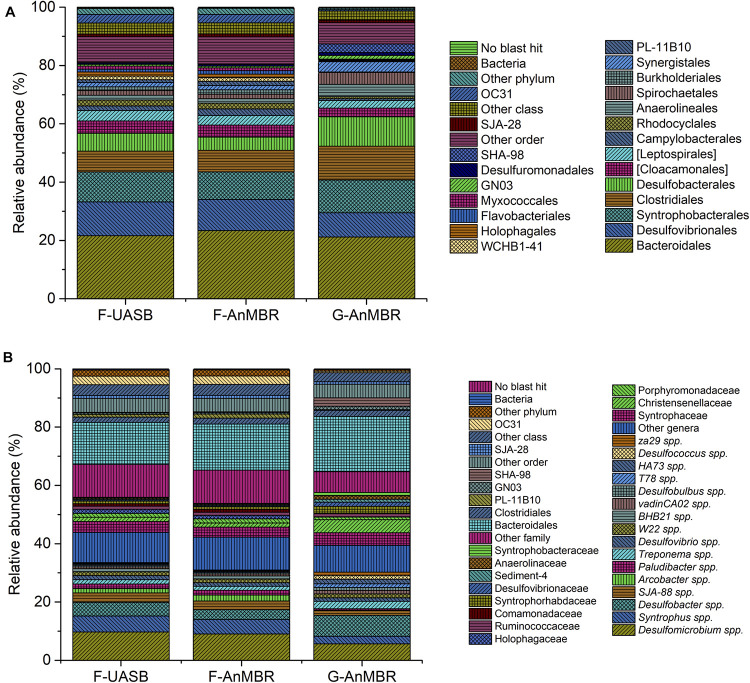
Taxonomic assignment of sequencing reads from bacterial community at 85% confidence level (order) **(A)** and at 95% confidence level (genera) **(B)**. Relative abundance was defined as the number of reads (sequences) affiliated with that taxon divided by the total number of reads per sample. Phylogenetic groups with relative abundance lower than 1% were categorised as “others”.

*Proteobacteria*, *Bacteroidetes*, and *Firmicutes* phyla accounted for an abundance of around 70% in the three reactors although its distribution was slightly different. In the three reactors, *Proteobacteria* was the predominant phylum, followed by *Bacteroidetes* and *Firmicutes*. Flocculent reactors presented slightly higher percentages for *Proteobacteria* (F-UASB—38%, F-AnMBR—36%, G-AnMBR—34%) and *Bacteroidetes* (F-UASB—23%, F-AnMBR—25%, G-AnMBR—21%) than the granular reactor. On the other hand, *Firmicutes* abundance was higher in the granular reactor (F-UASB—8%, F-AnMBR—8%, G-AnMBR—15%). The first step in the anaerobic digestion process is the hydrolysis of complex polymers to oligomers performed by hydrolytic fermentative bacteria. *Bacteroidetes* and *Firmicutes*, with a high level of metabolic diversity, are typically the predominant phyla of hydrolytic bacteria in anaerobic digestion as reviewed by Azman et al. (2015). In the three reactors, most of *Firmicutes* OTUs detected belonged to *Clostridiales* order (Figure 8), a group known for its capabilities in organic decomposition and fermentation (Desvaux, 2005). Also, microorganisms of the order *Bacteroidales*, belonging to the phylum *Bacteroidetes*, were the most predominant (F-UASB—22%, F-AnMBR—23%, G-AnMBR—21%) (Figure 8). *Bacteroidales* are known for saccharolytic and proteolytic activity and are capable of producing propionate, acetate and succinate while *Clostridiales* are involved in hydrolysis and the fermentation of carbohydrates (Ju et al., 2017; Buettner and Noll, 2018). Ju et al. (2017) suggested the codominance of *Bacteroidales* and *Clostridiales* given their different ecological traits and rules, occupying different niches in anaerobic digestion communities.

*Proteobacteria* abundance in anaerobic digesters is usually lower than the amount detected in the reactors studied in this work. The main orders detected within *Proteobacteria* included syntrophic bacteria (i.e., *Syntrophobacterales*) and sulfate reducing bacteria (SRB) (i.e., *Desulfovibrionales* or *Desulfobacterales*), as can be observed in Figure 8A. In anaerobic digestion, syntrophic organic acid degradation is crucial for stable wastewater treatment, given that acid accumulation is known to trigger acidification and process failure (Narihiro et al., 2015). Syntrophic bacteria, such as *Syntrophus* spp. or vadinCA02 spp. (Figure 8B), are capable of degrading organic matter to volatile fatty acids and hydrogen. *Syntrophus* spp. produce H_2_ through fermentation of organic compounds, being capable of maintaining syntrophic interactions with hydrogenotrophic methanogens (Botello Suárez et al., 2018). However, methanogens and SRB are hydrogen and acetate consuming organisms which contribute to the syntrophic relationship as consumers. Despite the high relative abundance of SRB detected in the samples (as can be observed from Figure 8B), methane production was steady during operation (Figure 3), thus, high abundance of SRB probably did not hamper methanogenesis mediated by syntrophic bacteria. Fed wastewater had a low COD content with respect to SO_4_^2–^ content, which led to a COD/SO_4_^2–^ ratio of the wastewater feed near 0.5. Lu et al. (2017) reported that methanogenic archaea could out-compete sulfate reducers even at a low COD/SO_4_^2–^ ratio of 0.5 and stated that low COD/SO_4_^2–^ favored the sulfidogenesis process and diversified the microbial community inside the reactor. Their research proved the beneficial effect of sulfidogenesis in favoring sludge re-granulation when treating high sulfate methanolic wastewater.

The potential enteric human pathogen *Arcobacter*, which was detected in all samples (Figure 8B) has been previously reported as part of residue microbiota (Penfield, 2017). Residue populations associated with undigested feed wastewater have been commonly observed in anaerobic digesters, being more abundant in low-temperature digesters as reported by Mei et al. (2017).

**FIGURE 9:**
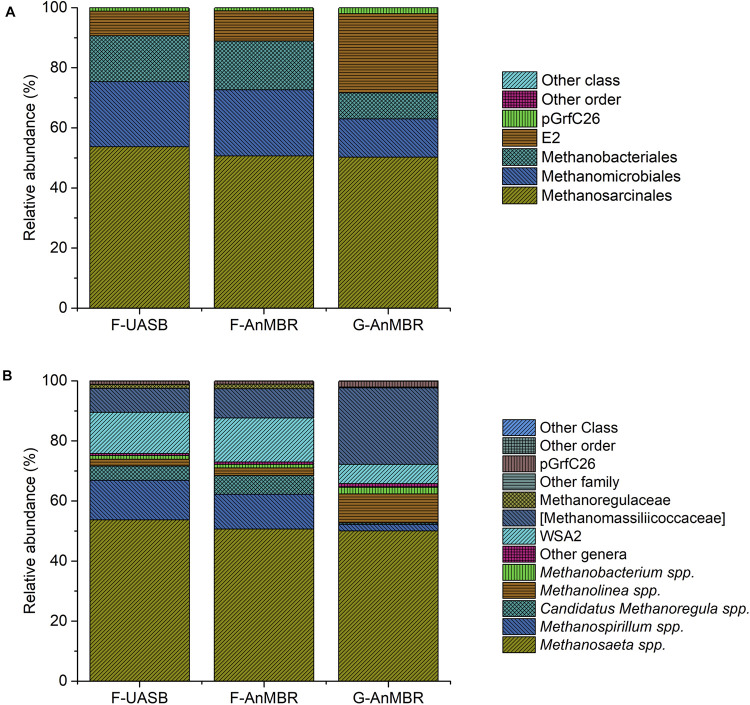
Taxonomic assignment of sequencing reads from archaeal community at 85% confidence level (order) **(A)** and at 95% confidence level (genera) **(B)**. Relative abundance was defined as the number of reads (sequences) affiliated with that taxon divided by the total number of reads per sample. Phylogenetic groups with relative abundance lower than 1% were categorised as “others”.

Archaea (Figure 9) are key organisms in anaerobic digestion processes, as they are responsible for methanogenesis step, in which CH_4_ is produced. There are three main types of methanogens according to the substrates used: acetoclastic (acetate), hydrogenotrophic (H_2_ and CO_2_), and methylotrophic (methylated compounds) although most of the CH_4_ is produced by the first two types. Only microorganisms of two genera are recognized as acetoclastic methanogens, *Methanosaeta* spp. and *Methanosarcina* spp. However, *Methanosarcina* spp. can use both acetate and H_2_. The most common genera within hydrogenotrophic methanogens are *Methanobacterium*, *Methanothermobacter*, *Methanobrevibacter*, *Methanospirillum*, and *Methanoculleus*. (Wang et al., 2018c).

In the three reactors, *Methanosaeta* spp. was by far the most abundant genus (F-UASB—54%, F-AnMBR—50%, G-AnMBR—50%) (Figure 9B). Thus, these results suggest the importance of the acetoclastic pathway when operating at low temperatures as took place in the current trial. However, relatively high abundance of hydrogenotrophic archaea (*Methanospirillum* spp., candidatus *Methanoregula* spp., *Methanolinea* spp., and *Methanobacterium* spp.) was also detected (Figure 9B), meaning probably both pathways coexisted in the reactors. Reviewed literature presents opposing results when it comes to the methanogenic pathway favored under psychrophilic conditions. On one hand, psychrophilic conditions have been described to favor hydrogenotrophic methanogenesis. *Methanomicrobiales* populations, and thus hydrogenotrophic methanogensis, have been reported to play an important role in low-temperature anaerobic granular sludge systems and digestion under psychrophilic conditions in a number of studies (McHugh et al., 2004; Connaughton et al., 2006; Zhang et al., 2012; Gunnigle et al., 2015; Tian et al., 2018). However, several authors also described an increase in acetoclastic methanogenesis and a high abundance of *Methanosarcinales* in anaerobic digestion under psycrophilic conditions (O’Reilly et al., 2009; Penfield, 2017; Zhang et al., 2018).

As can be observed in Figure 9A, *Methanomicrobiales* (F-UASB—22%, F-AnMBR—22%, G-AnMBR—13%) and *Methanobacteriales* (F-UASB—15%, F-AnMBR—16%, G-AnMBR—9%) were detected in higher abundance in the flocculent reactor while E2 (F-UASB—8%, F-AnMBR—10%, G-AnMBR—26%) presented a higher abundance in the granular reactor. *Methanomicrobiales* detected included *Methanospirillum* spp., candidatus *Methanoregula* spp., and *Methanolinea* spp. While the first two were more abundant in the flocculent reactors, *Methanolinea* spp. was more abundant in the granular reactor. Zhang et al. (2012) suggested *Methanomicrobiales* are likely to perform key roles in low-temperature anaerobic granular sludge systems and under psychrophilic conditions and also reported the detection of *Methanolinea* spp. at working temperatures of 5–18°C. Narihiro et al. (2015) reported *Methanolinea* spp. were specifically isolated by enrichment under syntrophic conditions. Group E2 (Figure 9A) was exclusively represented by candidatus *Methanomassiliicoccaceae* (Figure 9B) which has been reported to be an hydrogenotrophic methanogen (Iino et al., 2013). Therefore, from the obtained results, a coexistence of acetoclastic and hydrogenotrophic methanogenesis in the reactors is suggested.

In conclusion, the quick start-up of the reactors can be attributed to the previous acclimation of the biomass to the temperate treatment conditions, even with a 5-months inoperative period. The results obtained for the first 45 days of operation of the three different reactor configurations showed that solids management is critical for the anaerobic treatment of municipal wastewater using UASB reactors. Solids, colloids and particles need to be retained in the reactor to increase solids hydrolysis efficiency and thus, true separation processes such as membrane systems are necessary. Flocculent biomass promoted slightly higher hydrolysis than granular biomass possibly because flocculent sludge acts as a fine filter while granular acts as a coarse filter. From the results obtained, F-AnMBR showed a better performance for the treatment of municipal wastewater at 10°C. PCA analysis demonstrated that the microbial communities from the three reactors analyzed were highly similar. However, higher similarities were found in flocculent reactors, regardless of the membrane presence in the system. Thus, reactor inoculum had higher influence in microbial community than reactor configuration. *Proteobacteria*, *Bacteroidetes*, and *Firmicutes* phyla accounted for an abundance of around 70% in the three reactors although its distribution was slightly different. *Bacteroidales* and *Clostridiales* were the major bacterial fermenters orders detected and a relative high abundance of syntrophic bacteria, represented by *Syntrophobacterales*, was also detected. Additionally, an elevated abundance of SRB (i.e., *Desulfovibrionales* and *Desulfobacterales*) were identified and was attributed to the low COD/SO_4_^2–^ ratio of the wastewater. A coexistence of acetoclastic and hydrogenotrophic methanogenesis in the reactors is suggested given the high abundance of *Methanosaeta* spp. as well as *Methanomicrobiales and Methanobacteriales*.

## Data Availability Statement

The raw data supporting the conclusions of this article will be made available by the authors, without undue reservation.

## Author Contributions

JR-P completed all the experiments and drafted the manuscript. AC helped starting up the reactors and completed some of the wastewater analysis. MB-F advised on the manuscript data collection and provided minor comments on the manuscript. IJ advised on the manuscript data collection, experiment design, and provided comments on the manuscript. XM-L advised on the manuscript data collection, experiment design, and provided comments on the manuscript. BJ acted as an advisor on the project and provided minor comments on the manuscript. EM acted as an advisor on the project and provided minor comments on the manuscript. AS was the project principal investigator, having provided significant input on the data collection interpretation, writing of the manuscript, and completed all the revisions. All authors contributed to the article and approved the submitted version.

## Conflict of Interest

The authors declare that the research was conducted in the absence of any commercial or financial relationships that could be construed as a potential conflict of interest.
